# Case reports: combination therapy with proteasome inhibitor Bortezomib and humanized anti-CD25 Basiliximab for treatment of adult T cell leukaemia lymphoma

**DOI:** 10.1186/1742-4690-11-S1-P8

**Published:** 2014-01-07

**Authors:** Huseini Kagdi, Paul Fields, Andrew Hodson, Graham Taylor

**Affiliations:** 1 Infectious Diseases and Immunology, Faculty of Medicine, Imperial College, London, UK; 2 Department of Haematology, Guys Hospital, London, UK

## 

Adult T cell Leukaemia Lymphoma [ATL] is a mature T cell neoplasm affecting 2 - 6% of HTLV-1 infected subjects. ATL have been classified into 4 subtypes- smouldering, chronic leukaemia, acute leukaemia and lymphoma subtype. First line treatment with zidovudine (ZDV) and interferon-α (IFN) is recommended for smouldering, acute and chronic leukaemia whilst for lymphoma subtype combination chemotherapy is supplemented with ZDV/IFN. However a substantial proportion of patients do not respond or suffer treatment limiting side effects. Hence there is an urgent need for novel treatment options. Anti-CD25 in combination with bortezomib trialled in a murine ATLL model out performed either agent alone. We report 2 patients with Chronic ATL who were treated with a novel combination of humanized anti-CD25 antibody, Basiliximab and proteosome inhibitor, Bortezomib. Both patients had CD4+CD25+CCR4+CD127-lo ATL phenotype, were Interferon Regulatory Factor-4 negative and had responded to ZDV/IFN but had treatment limiting side effect. Prior to the novel combination baseline lymphocyte counts were 73.5 & 7.1 X 109/L, LDH 398 & 410 IU/L, CD4+CD25+ 97 & 97 % respectively. One patient had disseminated cutaneous lymphoma. Treatment was initially well tolerated and complete haematological remission was achieved after 8-14 weeks but flow cytometry revealed persistence of cells with the aforementioned ATLL phenotype. Discontinuation of Bortezomib in one patient, due to neuropathy, was associated with an immediate increase in CD4 counts. These early data suggest that this combination presents a new targeted therapy option for remission induction in treatment intolerant patients esp. leukemic subtype and warrant further clinical investigation.

